# Diagnostic Accuracy of Combined 3.0T Magnetic Resonance Imaging and Molybdenum Target X-Ray in Triple-Negative Breast Cancer: Correlation with Prognosis in Patients Undergoing Sentinel Lymph Node Biopsy

**DOI:** 10.1089/whr.2023.0080

**Published:** 2024-07-08

**Authors:** Li Xia, Ling Yang, Meng Hu

**Affiliations:** Shanghai Ninth People’s Hospital Affiliated to Shanghai Jiao Tong University School of Medicine, Huangpu, China.

**Keywords:** 3.0T magnetic resonance imaging, Molybdenum target X-ray, Triple-negative breast cancer, Diagnosis, Prognosis, Correlation

## Abstract

**Objective:**

This study assessed the diagnostic efficacy of combining 3.0T MRI and molybdenum target X-ray in triple-negative breast carcinoma (TNBC) and its association with the prognosis of sentinel lymph node biopsy (SLNB).

**Methods:**

The retrospective analysis included 128 patients suspected of having TNBC, who underwent 3.0T MRI and molybdenum target X-ray. Sensitivity and specificity were calculated for each imaging technique, and their combined diagnosis was evaluated using the four-table method. Consistency between the imaging techniques and pathological examination was assessed using the consistency checking method. Additionally, changes in imaging indicators were compared among patients with different prognostic indicators.

**Results:**

Among the 128 patients, 86 were diagnosed with TNBC through pathological examination. The sensitivity and specificity of 3.0T MRI for TNBC were 82.56% and 76.19%, respectively. Molybdenum target X-ray exhibited a sensitivity of 77.91% and specificity of 78.57%. The combined diagnosis of the two techniques showed a sensitivity of 90.70% and specificity of 86.36%. There was good agreement between both imaging techniques and pathological examination results. Significant differences were observed in imaging indicators based on tumor diameter, histological grade, and lymph node metastasis.

**Conclusion:**

Both 3.0T MRI and molybdenum target X-ray are valuable in diagnosing TNBC. Additionally, these imaging techniques provide prognostic information and can aid in treatment decision-making. The findings highlight the importance of 3.0T MRI and molybdenum target X-ray in improving the outcomes of patients with TNBC.

## Introduction

Breast carcinoma is a common malignant tumor among women worldwide. In China, it has become the second most prevalent female malignancy, and its incidence is increasing in younger women, thereby posing a serious public health concern.^1,2^ Triple-negative breast carcinoma (TNBC), which accounts for approximately 15–20% of all newly diagnosed breast carcinomas, is a distinct type of breast cancer characterized by the negative expression of estrogen receptor (ER), progesterone receptor (PR), and human epidermal growth factor receptor 2 (HER2) on immunohistochemical examination.^3,4^ TNBC is associated with strong invasiveness, poor prognosis, and high grade, and accounts for 5% of cancer-related deaths. Therefore, early diagnosis and effective treatment of TNBC are crucial for improving patient outcomes.

3.0T magnetic resonance imaging (3.0T MRI) is a high-field magnetic resonance imaging technique that provides high tissue resolution and can clearly display internal tumor changes, enabling evaluation of benign and malignant lesions.^5,6^ Molybdenum target X-ray examination is a commonly used clinical imaging technique for diagnosing breast diseases, where mass, calcification, and lesion density are the main basis for the clinical diagnosis of breast carcinoma.^7,8^ Although these techniques are effective for diagnosing breast carcinoma, there is limited research on their relationship with the prognosis of TNBC patients.

In this study, we retrospectively selected 86 TNBC patients admitted to our hospital between January 2019 and January 2021 as the research subjects to analyze the diagnostic efficacy of combining 3.0T MRI and molybdenum target X-ray in TNBC and its association with the prognosis of patients treated with sentinel lymph node biopsy.

## Data and Methods

### Study population

The study retrospectively analyzed case data from 128 patients with suspected TNBC who were admitted to our hospital between January 2019 and January 2021. These patients were considered as the study subjects. There is diagnostic uncertainty in these patients on initial imaging or clinical presentation, and at one time we considered 3T MRI and mammography as complementary diagnostic tools to increase diagnostic confidence and guide treatment decisions. Based on the results of pathological examination, the patients were divided into two groups: the TNBC group and the benign breast lesion group. The inclusion criteria for the TNBC group were as follows: ① Patients receiving initial treatment and diagnosed with breast carcinoma through pathological examination; ② Negative expression of ER, PR, and HER2 in immunohistochemical examination of cancer tissues; ③ Undergoing 3.0T MRI and molybdenum target X-ray examination prior to surgery; ④ Have good compliance and cooperate with examination and treatment. The exclusion criteria were: ① Patients with concomitant malignant tumors; ② Patients with severe dysfunction of vital organs; ③ Patients with nervous system or psychiatric diseases; ④ Patients with poor imaging quality or loss of images; ⑤ Lactating or pregnant women.

### Methods

3.0T MRI examination: The imaging examinations were conducted using a 3.0T Signa HDxt magnetic resonance imaging system. Patients were positioned in the prone position, allowing their breasts to naturally sag. Routine examinations were performed from coronal, sagittal, and transverse positions. The scanning sequence included T1-weighted imaging (T1WI), T2-weighted imaging (T2WI), dynamic contrast-enhanced MRI (DCE-MRI), and diffusion-weighted imaging (DWI) examinations. Prior to the DCE-MRI examination, intravenous access was established using selenate at a dose of 0.2 mmol/kg, with a speed of 3.0 ml/s. Continuous scanning was performed 15–25 seconds after injection. The scanning parameters included an 8 ms recurrence time (TR), 3.93 ms echo time (TE), 0.9mm0.8mm0.8 mm voxel size, 12 flips, 448 resolution, 34 mm field of view (FOV), 72 layers, 0.8 mm layer thickness, and a single scan time of 30 seconds. The capacity transfer constant (Ktrans) and reflux rate constant (Kep) values were measured and calculated at primary tumor site.

Molybdenum target X-ray examination: A HAWK-2M high-frequency mammography X-ray machine was used for molybdenum target X-ray examination. Patients were positioned in the supine position, and X-rays were taken from the head to the tail. X-rays were also taken from the parallel line of the scapula to the diaphragm. Calcifications, areas of high density, and structural distortion were observed and compared.

### Statistical methods

SPSS 20.0 software was used for analyzing the experimental data. (Σ*x* ± *s*) was used to represent age, axillary mass diameter, Kep and other measurement data, and *t-test* was used. Counting data such as lesion location, 3.0T MRI, and detection of molybdenum target X-ray were expressed in the form of (%), and the *χ*^2^ test was used. The sensitivity and specificity of 3.0T MRI, molybdenum target X-ray and combined diagnosis for TNBC were detected by using the four-table method. The consistency checking method was used to detect the consistency of 3.0T MRI, molybdenum target X-ray examination separately and the combination of the two and the pathological examination in diagnosing TNBC. The coefficient of internal consistency (Inter-rater, coefficient of internal consistency, Kappa) ≥0.75 indicated that the consistence between the two was better. Statistical results were considered statistically significant when *p* < 0.05.

## Results

### Analysis of the diagnostic values of 3.0T MRI in TNBC

The TNBC group consisted of 86 cases, with 56 cases on the left side and 30 cases on the right side. The average age was (52.12 ± 7.17) years old, and the average primary lesion diameter was (3.25 ± 0.63) cm. The benign breast lesion group included 42 cases, with 26 cases on the left side and 16 cases on the right side. The average age was (51.93 ± 6.43) years old, and the average axillary mass diameter was (3.19 ± 0.85) cm. There were no significant differences in terms of age, lesion location, and axillary mass diameter between the two groups (*p* > 0.05). The 86 TNBC patients were categorized based on prognostic indicators such as the largest tumor diameter, histological grade, and lymph node metastasis. Please refer to Table 1 for details.

**Table 1.** Analysis of General Data of the 2 Groups [n (%) (Σ*x* ± *s*)]

Group	n	Age(years)	Lesion location		Axillary lump diameter (cm)
			Left side	Right side	
TNBC group	86	52.12 ± 7.17	56 (65.12)	30 (34.88)	3.25 ± 0.63
Benign breast lesion group	42	51.93 ± 6.43	26 (61.90)	16 (38.10)	3.19 ± 0.85
*x* ^2^ */t*		0.144	0.126		0.428
*P*		0.886	0.722		0.670

Among the 128 patients in this experiment, 86 patients were diagnosed with TNBC by pathological examination, and the sensitivity and specificity of 3.0T MRI in the diagnosis of TNBC detected by the four-table method were 82.56% and 76.19%, respectively; the Kappa of 3.0T MRI and pathological examination in the diagnosis of TNBC detected by consistency checking method was 0.775, and the consistency between the two was good (*p* < 0.05). See Table 2.

**Table 2.** Analysis of the Diagnostic Values of 3.0T MRI in TNBC [n (%)]

Check method	Pathological examination		Total	Sensitivity	Specificity	Kappa	P
	Positive	Negative					
3.0T MRI							
Positive	71 (55.47)	10 (7.81)	81 (63.28)	82.56%	76.19%	0.775	<0.001
Negative	15 (11.72)	32 (25.00)	47 (36.72)				
Total	86 (67.19)	42 (32.81)	128 (100.00)				

### Analysis of the diagnostic values of molybdenum target X-ray in TNBC

The sensitivity and specificity of molybdenum target X-ray in the diagnosis of TNBC detected by four-table method were 77.91% and 78.57%, respectively; the Kappa of the molybdenum target X-ray and pathological examination in diagnosing TNBC detected by consistency checking method was 0.764, and the consistency between the two was good (*p* < 0.05). See Table 3.

**Table 3.** Analysis of Diagnostic Values of Molybdenum Target X-Ray in TNBC [n (%)]

Check method	Pathological examination		Total	Sensitivity	Specificity	Kappa	P
	Positive	Negative					
Molybdenum target X-ray							
Positive	67 (52.34)	9 (7.03)	86 (67.19)	77.91%	78.57%	0.764	<0.001
Negative	19 (14.84)	33 (25.78)	42 (32.81)				
Total	86 (67.19)	42 (32.81)	128 (100.00)				

### Analysis of the diagnostic values of 3.0T MRI combined with molybdenum target X-ray in TNBC

The sensitivity and specificity of 3.0T MRI combined with molybdenum target X-ray in the diagnosis of TNBC detected by the four-table method were 90.70% and 86.36%, respectively; the Kappa of 3.0T MRI combined with molybdenum target X-ray and pathological examination in the diagnosis of TNBC detected by consistency checking method was 0.836, and the consistency between the two was good (*p* < 0.05). See Table 4.

**Table 4.** Analysis of the Diagnostic Values of 3.0T MRI Combined with Molybdenum Target X-Ray in TNBC [n (%)]

Check method	Pathological examination		Total	Sensitivity	Specificity	Kappa	P
	Positive	Negative					
3.0TMRI+X-ray							
Positive	78 (60.94)	6 (4.69)	84 (65.63)	90.70%	86.36%	0.836	<0.001
Negative	8 (6.25)	36 (28.13)	44 (34.38)				
Total	86 (67.19)	42 (32.81)	128 (100.00)				

### Correlation analysis between 3.0T MRI indicators and TNBC prognosis

**FIG. 1.**
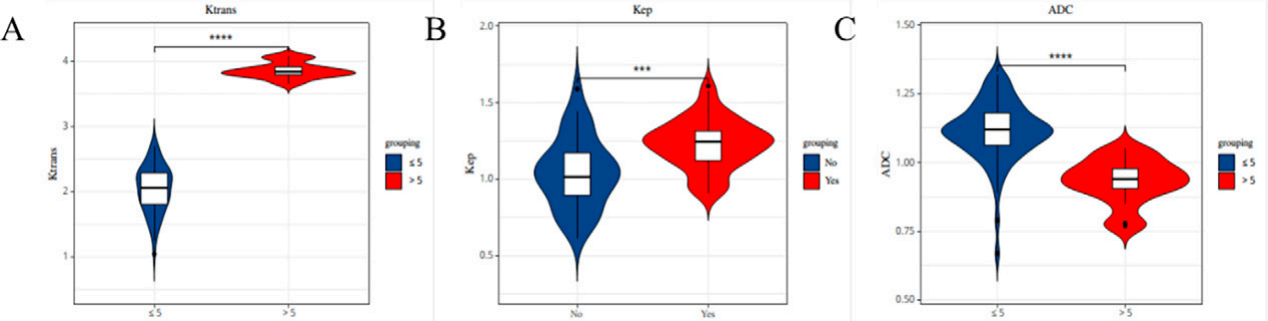
Analysis of the relationship between 3.0T MRI indicators and the largest diameter of TNBC tumors. **(A)** is the analysis of the relationship between 3.0T MRI Ktrans indexes and the largest diameter of TNBC tumors; **(B)** is the analysis of the relationship between 3.0T MRI Kep indexes and the largest diameter of TNBC tumors; **(C)** is the analysis of the relationship between 3.0T MRI ADC indexes and the largest diameter of TNBC tumors.

**FIG. 2.**
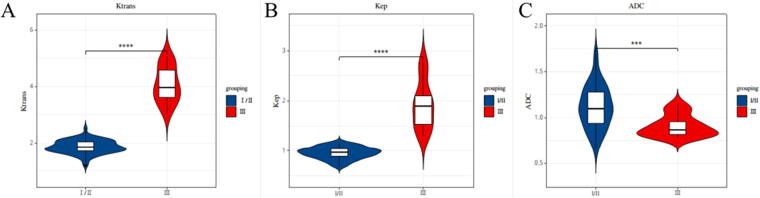
Analysis of the relationship between 3.0T MRI indicators and TNBC histological grade. **(A)** is the analysis of the relationship between 3.0T MRI Ktrans indexes and TNBC histological grade; **(B)** is the analysis of the relationship between 3.0T MRI Kep indexes and TNBC histological grade; **(C)** is the analysis of the relationship between 3.0T MRI ADC indexes and TNBC histological grade.

**FIG. 3.**
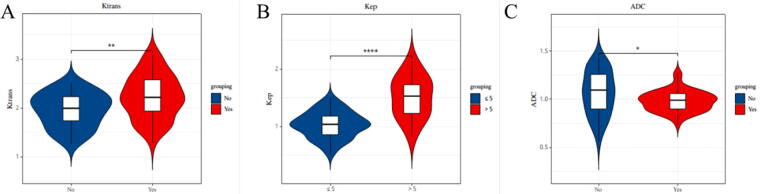
Analysis of the relationship between 3.0T MRI indicators and TNBC lymph node metastasis. **(A)** is the analysis of the relationship between 3.0T MRI Ktrans indexes and TNBC lymph node metastasis; **(B)** is the analysis of the relationship between 3.0T MRI Kep indexes and TNBC lymph node metastasis; **(C)** is the analysis of the relationship between 3.0T MRI ADC indexes and TNBC lymph node metastasis.

The levels of Ktrans and Kep in the group with the largest tumor diameter > 5 cm were obviously greater than that of the group with the largest tumor diameter ≤5 cm, and the level of ADC was obviously less than that in the group with the largest tumor diameter ≤5 cm; the levels of Ktrans and Kep in histological grade III group were obviously greater than those in histological grade I/II group and the level of ADC was obviously less than that in the histological grade I/II group; the levels of Ktrans and Kep in the lymph node metastasis group were obviously greater than those in the lymph node non-metastasis group, and the distinction had statistical significance (*p* < 0.05). See Table 5, Figs. 1, 2, and 3.

**Table 5.** Correlation Analysis between 3.0T MRI Indicators and TNBC Prognosis (Σ*x* ± *s*)

Group	n	Ktrans	Kep	ADC (×10^−3^ mm^2^ /s)
The largest tumor diameter				
≤5	70	2.04 ± 0.36	1.03 ± 0.23	1.11 ± 0.11
>5	16	3.86 ± 0.12	1.51 ± 0.36	0.93 ± 0.81
*t*		34.529	5.064	6.078
*P*		<0.001	<0.001	<0.001
Histological grade				
Grade I/II	71	1.86 ± 0.23	0.96 ± 0.12	1.12 ± 0.23
Grade III	15	4.08 ± 0.63	1.92 ± 0.48	0.91 ± 0.12
*t*		13.485	7.725	5.117
*P*		<0.001	<0.001	<0.001
Lymph node metastasis				
Not transferred	34	1.96 ± 0.35	1.04 ± 0.23	1.06 ± 0.23
Transferred	52	2.23 ± 0.45	1.23 ± 0.16	0.98 ± 0.11
*t*		3.004	4.120	1.923
*P*		0.004	<0.001	0.061

### Correlation analysis of molybdenum target X-ray imaging features and TNBC prognosis

The proportions of high-density of diseased area and structural distorted signs of mammography X-ray in the tumor maximum diameter > 5 cm group were obviously greater than that of the tumor maximum diameter ≤ 5 cm group; the proportions calcification and structural distorted signs of mammography X-ray in the histological grade III group were obviously greater than that of histological grade I/II group; the proportions of high density of diseased lesion and structural distorted signs of the molybdenum target X-ray in the lymph node metastasis group were obviously greater than those in the lymph node non-metastasis group (*p* < 0.05). See Table 6.

**Table 6.** Correlation Analysis between Molybdenum Target X-Ray Imaging Feature and TNBC Prognosis [n (%)]

Time	Group	n	Calcification (*n* = 47)	High density of diseased area (*n* = 57)	Structural distortion (*n* = 40)
Tumor diameter	≤5	70	38 (54.29)	43 (61.43)	28 (40.00)
	>5	16	9 (56.25)	14 (87.50)	12 (75.00)
*x* ^2^			0.020	3.961	6.422
*P*			0.887	0.047	0.011
Histological grade	Grade I/II	71	35 (49.30)	45 (63.38)	29 (40.85)
	Grade III	15	12 (80.00)	12 (80.00)	11 (73.33)
*x* ^2^			4.711	1.531	5.254
*P*			0.030	0.216	0.022
Lymph node metastasis	Not transferred	34	17 (50.00)	10 (29.41)	10 (29.41)
	Transferred	52	30 (57.69)	47 (90.38)	30 (57.69)
*x* ^2^			0.491	34.196	6.609
*P*			0.484	<0.001	0.010

## Discussion

Triple-negative breast cancer (TNBC) is characterized by high proliferative activity, rapid growth, and strong invasiveness. The disease has a short clinical course, high incidence of early metastasis, and poor prognosis.^9^ Therefore, early clinical diagnosis and aggressive surgical and systemic treatments are crucial for improving patient outcomes.

In recent years, medical imaging technology has rapidly advanced and is widely used in clinical practice worldwide. Among these technologies, X-ray mammography is commonly used for the clinical diagnosis of breast carcinoma due to its high detection rate and accuracy. It aids in early diagnosis and guides treatment planning.^10,11^ Molybdenum target X-ray, as an emerging technology, offers automatic adjustment for imaging and intelligent inspection. It is characterized by simple operation, fast inspection speed, and improved contrast and image quality. This technology helps doctors evaluate benign and malignant lesions more accurately.^12,13^

3.0T MRI, which includes T1WI, T2WI, DCE-MRI, and DWI, provides extremely high-quality images. Compared to conventional MRI, 3.0T MRI offers higher definition and resolution, enabling accurate evaluation of benign and malignant changes in lesions.^14,15^ Furthermore, 3.0T MRI utilizes electromagnetic waves for imaging without causing significant harm or radiation exposure. Random sections are imaged and scanned during the inspection process, allowing for more comprehensive assessment of lesion sites. This improves the detection rate and diagnosis of benign and malignant lesions.^16,17^

In this study, the sensitivity and specificity of 3.0T MRI in diagnosing TNBC were 82.56% and 76.19%, respectively. The Kappa coefficient for diagnosing TNBC through pathological examination was 0.775. For molybdenum target X-ray, the sensitivity and specificity in diagnosing TNBC were 77.91% and 78.57%, respectively. The Kappa coefficient for diagnosing TNBC through pathological examination was 0.764. When used in combination, 3.0T MRI and molybdenum target X-ray achieved a sensitivity and specificity of 90.70% and 86.36%, respectively. The Kappa coefficient for combined diagnosis and pathological examination in diagnosing TNBC was 0.836, indicating excellent consistency between the two methods. These results demonstrate that both 3.0T MRI and molybdenum target X-ray have value in the diagnosis of TNBC, aiding in the preoperative evaluation of benign and malignant breast lesions. Combining these imaging modalities enhances diagnostic accuracy, facilitating early diagnosis and intervention, thus improving patient prognosis.

3.0T MRI provides high-resolution images of soft tissues, accurately displaying hemodynamic changes in lesions.^18,19^ Diffusion-weighted imaging (DWI), based on the Brownian motion of water molecules, evaluates changes in tissue structures by analyzing the speed of water molecule diffusion within lesions. Lower apparent diffusion coefficient (ADC) levels indicate higher cell density, increased risk of lesion malignancy, and worse patient prognosis.^20,21^ Dynamic contrast-enhanced MRI (DCE-MRI) reflects microcirculatory blood perfusion in lesion tissues. It is non-invasive and highly accurate.^22,23^ Among DCE-MRI parameters, Ktrans represents the transfer constant of contrast agent diffusion from blood vessels to extracellular space, while Kep indicates the ratio constant of contrast agent transport from extravascular extracellular space to blood vessels. Higher Ktrans and Kep levels imply increased extracellular space penetration and greater malignancy of the lesion.^24–27^

In this study, the Ktrans and Kep levels were significantly higher in the tumor diameter > 5 cm group, histological grade III group, and lymph node metastasis group compared to the tumor maximum diameter ≤ 5 cm group, histological grade I/II group, and lymph node non-metastasis group. Furthermore, ADC levels were significantly lower in the tumor maximum diameter ≤ 5 cm group, histological grade I/II group, and lymph node non-metastasis group. These findings indicate that changes in 3.0T MRI indicators are closely related to the prognosis of TNBC patients after treatment. This is consistent with the research by Kang SR et al., which demonstrated that triple-negative breast carcinomas exhibit higher Ktrans and kep levels than luminal carcinomas. Higher Ktrans and kep levels on DCE-MRI are associated with histopathological factors indicating poor prognosis. Therefore, DCE-MRI perfusion parameters can serve as useful imaging biomarkers for assessing tumor prognosis and angiogenesis. Regular monitoring of changes in DCE-MRI perfusion parameters can help predict patient outcomes.

Molybdenum target X-ray images primarily show characteristics such as calcification, boundary changes, and density variations. Some researchers believe that there is a certain relationship between these features and the changes and prognosis of TNBC.^28^ The experimental study found that the proportions of high density and structurally distorted signs in molybdenum target X-ray images were significantly higher in the group with a tumor diameter > 5 cm, histological grade III group, and lymph node metastasis group compared to the respective control groups. These findings suggest that molybdenum target X-ray image performance is also related to the prognostic indicators of TNBC patients, aiding in prognosis prediction.

Hormone-sensitive breast masses, such as estrogen receptor-positive (ER+) or progesterone receptor-positive (PR+) tumors, have different characteristics and treatment options compared to TNBC. Diagnostic imaging plays a crucial role in determining the nature of the mass, guiding treatment decisions, and assessing prognosis. Therefore, analyzing the performance of MRI and targeted X-ray in hormone-responsive tumors is indeed necessary to provide a comprehensive understanding of their diagnostic value.MRI has been widely used for the evaluation of hormone-sensitive breast masses. It provides detailed information about tumor morphology, size, and invasion into surrounding tissues. Additionally, dynamic contrast-enhanced MRI (DCE-MRI) can assess the vascularity and perfusion of the mass, which can be indicative of tumor aggressiveness. The high-resolution images obtained from MRI help in distinguishing malignant masses from benign lesions and aid in surgical planning.^1^

As for targeted X-ray imaging, while it primarily reveals features such as calcification, boundary changes, and density alterations, it can still provide valuable information for hormone-responsive tumors. For instance, the presence of microcalcifications on X-ray images can be suggestive of early-stage ductal carcinoma *in situ* (DCIS), which is often associated with ER+ tumors^2^. X-ray imaging also helps evaluate the extent of disease involvement, particularly in cases where there is calcification beyond the primary mass.By combining the information obtained from MRI and targeted X-ray imaging, clinicians can gain a more comprehensive understanding of hormone-responsive breast masses. The integration of imaging findings with clinical and pathological data allows for better treatment planning, including neoadjuvant endocrine therapy or targeted therapies, which are often used in the management of ER+ or PR+ tumors.

To make accurate recommendations about the routine use of these imaging modalities, further research and analysis are needed to assess their performance specifically in hormone-responsive breast masses. This involves evaluating the sensitivity, specificity, and overall diagnostic accuracy of MRI and targeted X-ray in distinguishing benign from malignant hormone-sensitive tumors and assessing their impact on treatment decisions and patient outcomes.

In conclusion, both 3.0T MRI and mammography X-ray have value in the diagnosis of TNBC, with high diagnostic accuracy. Additionally, there is a correlation between 3.0T MRI indicators, certain molybdenum target X-ray indicators, and prognostic factors of breast cancer. These findings contribute to early prognosis assessment and treatment guidance, ultimately improving patient outcomes.
